# External Validation of a risk stratification model to assist shared decision making for patients starting renal replacement therapy

**DOI:** 10.1186/s12882-016-0253-3

**Published:** 2016-04-07

**Authors:** Patrick Peeters, Wim Van Biesen, Nic Veys, Wim Lemahieu, Bart De Moor, Johan De Meester

**Affiliations:** Renal Division, Department Of Internal Medicine, Ghent University Hospital, Ghent, Belgium; Renal Division, Imelda Ziekenhuis, Bonheiden, Belgium; Renal Division, Jessa Hospital, Hasselt, Belgium; Renal Division, AZ Nikolaas, St-Niklaas, Belgium

## Abstract

**Background:**

Shared decision making is nowadays acknowledged as an essential step when deciding on starting renal replacement therapy. Valid risk stratification of prognosis is, besides discussing quality of life, crucial in this regard. We intended to validate a recently published risk stratification model in a large cohort of incident patients starting renal replacement therapy in Flanders.

**Methods:**

During 3 years (2001–2003), the data set collected for the Nederlandstalige Belgische Vereniging voor Nefrologie (NBVN) registry was expanded with parameters of comorbidity. For all incident patients, the abbreviated REIN score(aREIN), being the REIN score without the parameter “mobility”, was calculated, and prognostication of mortality at 3, 6 and 12 month after start of renal replacement therapy (RRT) was evaluated.

**Results:**

Three thousand four hundred seventy-two patients started RRT in Flanders during the observation period (mean age 67.6 ± 14.3, 56.7 % men, 33.6 % diabetes). The mean aREIN score was 4.1 ± 2.8, and 56.8, 23.1, 12.6 and 7.4 % of patients had a score of ≤4, 5–6, 7–8 or ≥9 respectively. Mortality at 3, 6 and 12 months was 8.6, 14.1 and 19.6 % in the overall and 13.2, 21.5 and 31.9 % in the group with age >75 respectively. In RoC analysis, the aREIN score had an AUC of 0.74 for prediction of survival at 3, 6 and 12 months. There was an incremental increase in mortality with the aREIN score from 5.6 to 45.8 % mortality at 6 months for those with a score ≤4 or ≥9 respectively.

**Conclusion:**

The aREIN score is a useful tool to predict short term prognosis of patients starting renal replacement therapy as based on comorbidity and age, and delivers meaningful discrimination between low and high risk populations. As such, it can be a useful instrument to be incorporated in shared decision making on whether or not start of dialysis is worthwhile.

**Electronic supplementary material:**

The online version of this article (doi:10.1186/s12882-016-0253-3) contains supplementary material, which is available to authorized users.

## Background

Worldwide, age at inception of renal replacement therapy is increasing. In Europe, the median age at start of RRT has increased from 64 to 73 years over the last decade [1]. Whereas start of renal replacement therapy can be lifesaving, it is associated with a high short term mortality and a substantial decrease in quality of life for some patients [2, 3]. In this setting, there is an increasing interest for the concept of conservative care [4–6], as it is accepted that for some patients the benefits of starting renal replacement therapy do not outweigh the drawbacks. Existing literature indicates that mere age on itself is insufficient to prognosticate outcome after start of RRT, and that rather presence of comorbidities should be taken into account [7–9].

Studies indicate that physicians tend to be overly optimistic on the prognosis of their patients [10]. Such a failure to recognise a poor prognosis might lead to perseveration of therapy and overemphasis of cure rather than care [5, 11] and deprive patients from achieving a good and serene death [4].

In order to discuss the option of conservative care with patients, there is a need for risk stratification models that can more objectively quantify prognosis. Such models should be valid from the statistical point of view, not only within the test population, but also in other external populations. Recently, a prognostic model was developed for 5 year mortality versus need for RRT in patients with CKD [12]. This model can be useful for longer term planning of patients with CKD, but is not intended to predict survival in patients with CKD stage 5, and cannot assist in decision making on whether to start RRT or opt for conservative care.

Observational studies consistently demonstrate that as comorbidity adds up, the survival advantage of starting renal replacement goes down, and survival between patients started on RRT and those with conservative care become similar [9, 13, 14]. In this regard, risk stratification models based on survival of patients who have actually started RRT can provide valid information in the decision making of whether or not starting RRT is warranted. Recently, such a model was published, based on data from the Renal Epidemiology Information Network (REIN) registry in France [15].

We intended to check the validity of this model in a large registry cohort of patients starting RRT in the Flanders region of Belgium, coordinated by the Nederlandstalige Vereniging voor Nefrologie.

## Methods

### Patients

The registry of the NBVN is a voluntary database which covers 100 % of all patients starting renal replacement therapy in Flanders, Belgium. As in the REIN database, patients with acute kidney injury are excluded.

The registry captures a basic set of baseline data through a web based system consisting of the following items : date of birth, gender, city/zip code, primary renal disease, date of first contact at the nephrology department, weight, length, serum creatinine at start of RRT, change of RTT modality (type and date), and mortality (cause and date).

Between 1/1/2001 until 31/12/2003, a more expanded set of data of comorbidities was collected at baseline, one and two years, with the intention to allow prognostic modelling. Baseline information at dialysis initiation included age, gender, eGFR based on creatinine and the MDRD formula, body mass index (BMI), serum albumin the month preceding dialysis start, diabetes (type 1 or 2), congestive heart failure (New York Heart Association stages I to IV), ischaemic heart disease (including history of myocardial infarction, coronary vascular disease, coronary artery bypass surgery, angioplasty or abnormal angiography), peripheral vascular disease (Leriche classification stages I to IV), cerebrovascular disease, arrhythmia, chronic obstructive pulmonary disease (COPD), malignancy, liver cirrhosis, mental disorders (defined to include dementia and psychosis), initial dialysis modality, and late referral (defined as starting dialysis less than 3 months after first contact with the nephrology department.

Patient data were entered by the individual centres and are validated on an ongoing basis by the registry data manager.

### Analysis

For each patient, the abbreviated REIN (aREIN) score was calculated based on the baseline comorbidity data as depicted in the original paper [15]. As the NBVN registry does not capture data on mobility, this item was omitted from the REIN score, and therefore, we designated this as the “abbreviated REIN score” (aREIN score) (Table 1).

**Table 1:** Parameters of the abbreviated REIN score

Risk factors	Points
Gender	
Male	1
Female	0
Age (years)	
[75–80]	0
[80–85]	0
[85–90]	2
> = 90	3
Congestive heart failure	
No	0
Stage I-II	2
Stage III-IV	4
Peripheral vascular disease	
No or stage I-II	0
Stage III-IV	1
Arrhythmia	
No	0
Yes	1
Cancer	
No	0
Yes	2
Severe behavioural disorder	
No	0
Yes	2
Serum Albumin (g/l)	
<25	5
[25–30]	3
[30–35]	2
≥35	0

The abbreviated REIN score is the REIN score without the “mobility topic”. In the original REIN score, patients get 0 points if they walk without help, 4 points if they need assistance for transfer and 9 points if they are totally dependent for transfer

Demographic data of patients were represented as mean ± standard deviation for continuous variables or n out of N (n/N) and percentages for categorical variables. Between group comparisons were done with One Way Analysis of Variance and post hoc testing with Least Square Difference for continuous variables, or Chi-square analysis for categorical variables.

Mortality rates at 3, 6 and 12 months were calculated, and this for four different severity stages of the aREIN score (low risk:≤4; moderate low risk: 5–6; moderate high risk: 7–8; and high risk: ≥9 points). Those four groups were selected as they represented a reasonable division of our cohort and resulted in subgroups with comparable mortality risk (see Additional file 1: Table S1).

Areas under the curve (AUC) were calculated for prediction of 3, 6 and 12 months survival based on the aREIN score.

Cox proportional hazards analysis was used to model survival in the different severity stages of the aREIN score.

All statistical analysis were performed in SPSS®version 22.

## Results

During the observation period, 3472 patients started renal replacement therapy. The demographic data of these patients are depicted in Table 2 according to their aREIN stage. For 793 patients (22.8 %) information on one parameter of the REIN score was missing, making aREIN score calculation impossible, leaving 2679 patients available for analysis. There was no difference in gender or age between those with versus without missing data.

**Table 2:** Demographic data of included patients, stratified according to the abbreviated REIN score

Abbreviated REIN score		≤4 *n* = 1523	5–6 *n* = 619	7–8 *n* = 338	≥9 *n* = 199	*p*-value
Screa at start (mg/dl)		7.2 ± 3.1	6.3 ± 2.7	6.4 ± 4.5	5.9 ± 3.1	<0.001
Weight (kg)		71.6 ± 15.7	71.6 ± 14.5	69.8 ± 14.5	69.3 ± 16.1	0.18
Height (cm)		166.5 ± 9.3	166.0 ± 8.1	168.5 ± 9.3	166.6 ± 9.1	0.21
Age at start (years)		64.0 ± 15.2	71.7 ± 11.0	77.5 ± 10.8	67.8 ± 14.3	<0.001
Female (%)		48.1	38.6	34.6	32.7	<0.001
Heart Failure	Stage 0–1	70.7	34.4	16.9	8.5	<0.001
	Stage 2	13.3	16.2	19.5	9.5	
	Stage 3	13.4	23.7	34.0	62.3	
	Stage 4	2.1	19.7	12.7	17.6	
Angina Pectoris	None	86.6	66.2	61.2	54.3	<0.001
	Grade 1	7.4	14.1	16.0	16.1	
	Grade 2	4.1	9.9	9.5	8.0	
	Grade 3	1.4	6.9	10.1	14.6	
Peripheral Vascular Disease						<0.001
None		87.2	71.9	70.4	61.3	
Claudicatio		8.1	15.2	15.7	17.6	
Resting ischemia		1.4	3.6	3.8	7.0	
Necrosis/amputation		3.2	9.4	10.1	14.1	
COPD	None	83.2	71.1	66.0	59.8	<0.001
	Mild	9.8	17.4	19.5	18.1	
	Moderate	5.6	8.6	9.8	14.1	
	Severe	1.4	2.9	4.7	8.0	
Myocardial Infarction		11	25.5	27.8	33.2	<0.001
Percutaneous Coronary Intervention		13	22.6	25.7	25.6	<0.001
Arrhythmia		6.4	18.4	28.7	44.2	<0.001
Diabetes		30.2	39.7	37.3	34.7	<0.001
Diabetic nephropathy		23	26.8	24.0	23.6	0.3
CerebroVascular Disease		8.7	8.7	13.7	15.0	<0.001
Dementia		0.7	2.4	7.4	8.5	<0.001
Liver disease		4.7	4.2	4.0	6.5	<0.001
Left ventricular Hypertrophy		49.5	51.5	51.2	54.8	0.6
Cancer		8.5	19.1	29.9	34.7	0.2
Nutritional status	Normal	92	75.4	60.9	49.2	<0.001
	Malnutrition	7.4	20.2	31.7	37.7	
	Severe malnutrition	0.6	4.4	13.1	13.1	
Albumin (g/l)	<25	0	18.3	27.2	45.2	<0.001
	25–30	5.6	18.3	27.5	31.2	
	30–35	19.2	33.4	30.5	22.1	
	35–40	49.6	22.0	9.8	1.0	
	>40	25.6	8.1	5.0	0.5	
C-reactive protein (mg/L)	<15	73.3	54.1	44.1	40.7	<0.001
	15–60	20.1	29.4	35.2	33.7	
	>60	6.6	16.5	20.7	25.6	
Renal Replacement Therapy						<0.001
	Hospital HD	77.7	80.9	85.8	88.4	
	Satellite HD	9.5	12.1	6.5	6.5	
	PD	12.8	6.9	7.7	4.5	

More than half (56.4 %) and almost three quarters (70.3 %) of those older than 85 and 90 years of age respectively at start of dialysis had an aREIN stage of 3 or 4.

We registered 276 (8.6 %), 453 (14.1 %) and 681 (19.6 %) deaths at 3, 6 and 12 months respectively.

Patients who died during the first 3 months were older (74.3 ± 9.9 vs 67.0 ± 14.5 years, *p* < 0.001), had a higher aREIN score at start (6.4 ± 2.7 vs 3.9 ± 2.7, *p* < 0.001), and a lower serum creatinine (6.1 ± 3.8 vs 6.7 ± 3.1 mg/dl, *p* < 0.01) and body weight (69.4 ± 15.5 vs 71.7 ± 15.7 kg, *p* = 0.03) as compared to those who survived (Table 3). Similar results were found for mortality at 6 and 12 months (results not shown).

**Table 3:** Comorbidities in patients in surviving vs dying within the first 3 months after start of RRT

		% in those who Survived	% in those who died	Risk estimate (95 % CI)
Age	<75	48.0	27.5	1.41
	75–80	17.4	16.7	(1.30–1.56)
	80–85	18.6	26.4	
	85–90	11.7	20.3	
	>90	4.3	9.1	
Gender	male	56.6	57.6	0.96
	female	43.4	42.4	(0.75–1.23)
Heart Failure	Stage 0	52.0	31.1	1.61
	Stage 1	14.3	11.2	(1.45–1.80)
	Stage 2	16.5	13.8	
	Stage 3	14.0	23.5	
	Stage 4	3.3	20.4	
Angina Pectoris	None	77.7	58.7	1.54
	Grade 1	10.4	14.8	(1.35–1.73)
	Grade 2	6.4	7.1	
	Grade 3	4.1	13.8	
	Grade 4	1.5	5.6	
Peripheral Vascular Disease	None	80.5	68.4	1.26
	Claudication	10.9	18.4	(1.08–1.47)
	Resting ischemia	2.4	5.6	
	Necrosis/amputation	6.2	7.7	
COPD	None	78.0	57.7	1.61
	Mild	12.6	24.5	(1.37–1.88)
	Moderate	7.2	10.7	
	Severe	2.3	7.1	
Myocardial Infarction	NO	82.5	74.0	1.66
	YES	17.5	26.0	(1.19–2.3)
PCI	NO	82.3	81.6	1.52
	YES	17.7	18.4	(0.72–1.54)
Arrhythmia	NO	85.7	78.6	1.64
	YES	14.3	21.4	(1.15–2.34)
Diabetes	NO	66.1	69.9	0.83
	YES	33.9	30.1	(0.61–1.15)
CerebroVascular Disease	NO	85.4	84.2	1.19
	YES	14.6	15.8	(0.90–1.56)
Dementia	NO	97.6	95.9	1.75
	YES	2.4	4.1	(0.82–3.70)
Liver disease	NO	95.1	94.9	1.05
	YES	4.9	5.1	(0.54–2.03)
Left ventricular Hypertrophy	NO	48.8	57.1	0.72
	YES	51.2	42.9	(0.54–0.96)
Cancer	NO	85.1	76.0	1.80
	YES	14.9	24.0	(1.27–2.55)
Nutritional status	Normal	83.3	53.1	3.06
	Malnutrition	14.2	34.7	(2.46–3.85)
	Severe malnutrition	2.5	12.2	
Albumin (g/L)	<25	10.0	24.0	1.67
	25–30	12.1	27.0	(1.48–1.87)
	30–35	24.3	21.9	
	35–40	35.7	19.9	
	≥40	17.9	7.1	
C-reactive protein (mg/L)	<15	65.6	27.6	2.76
	15–60	24.0	39.3	(2.29–3.31)
	≥60	10.4	33.2	
Renal Replacement Therapy	Hospital HD	78.9	87.0	1.78
	Satellite HD	9.2	6.2	(1.54–2.1)
	PD	11.3	6.9	

**Fig. 1:**
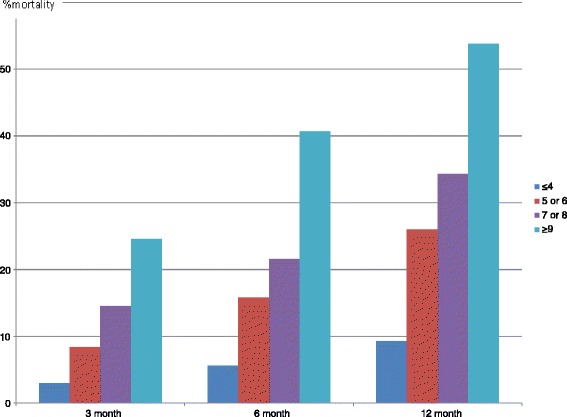
Mortality at 3, 6 and 12 months after start of RRT according to the stages of the abbreviated REIN score

Mortality at 3, 6 and 12 months according to 4 stages of aREIN score is graphically presented in Fig. 1, showing a good discrimination in mortality risk between the different aREIN stages. The results for the separate scores are presented in Additional file 1: Table S1.

Relative risk of death increased with each increasing aREIN stage with a factor 1.80 (95 % CI: 1.67–1.92), 1.78 (95 % CI: 1.64–1.96) and 1.80 (95 % CI: 1.61–1.99) at 3, 6 and 12 months respectively.

**Fig. 2:**
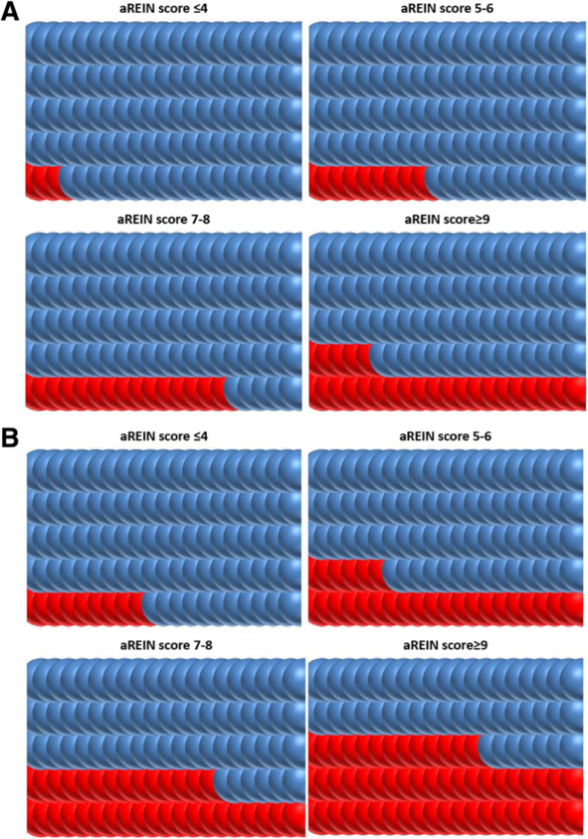
Survival according to aREIN stage panel A: 3 month survival; panel B: 12 month survival; Blue dots: patients who survived this period; Red dots: patients who did not survive this period

Figure 2 presents these survival data in the different aREIN stages in a format that facilitates shared decision making with patients.

Area under the curves for 3, 6 and 12 months mortality are 0.74 (95 % CI: 0.70–0.77), 0.74 (95%CI: 0.71–0.76) and 0.74 (95 % CI: 0.72–0.76).

**Fig. 3:**
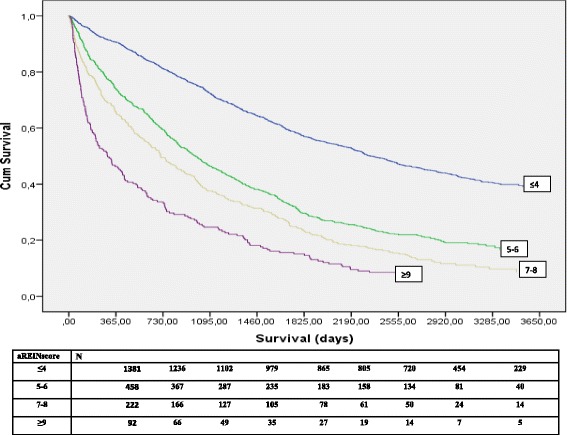
Long term survival after start of RRT in function of aREIN stage

Long term survival according to different stages of the aREIN score is depicted in Fig. 3, confirming that also longer term mortality risk goes up with aREIN stage.

## Discussion

This paper provides an external validation for a recently proposed risk stratification score for short term mortality in patients starting with renal replacement therapy [15]. In the NBVN registry, 3 month mortality risk ranged from 3 % in the lowest risk group to 24.6 % in the highest risk group. The score also performed well in discriminating risk at 6 and 12 months. In the highest risk stage, mortality at one year was higher than 50 %, whereas in the lowest risk stage, this was less than 10 %. With each increase in the aREIN stage, the relative risk for death at 3, 6 or 12 month increased with a factor 1.8. The aREIN score is based on easily available items and can be calculated by hand during outpatient consultation. Items included in the score are all well-acknowledged risk factors that are generally registered in the patient file under one format or another, but are rarely considered together to construct a global picture of the patient. Therefore, the calculation of the risk stratification score can help to increase a more holistic awareness on the prognostic risk of a patient and to identify patients at risk for a dismal outcome. The visual representation of the risk of mortality after start of RRT as depicted in Fig. 2 can help in the shared decision making on whether to start renal replacement therapy or to opt for conservative care.

Over the last decade, the age and comorbidity of patients starting renal replacement therapy has steadily increased [1]. In Flanders for example, the median age at start increased from 67.7 years in 1997 to 74.7 years in 2013. Over this period, the percentage of patients >75 years of age increased from 22.8 to 36.7 %, while over the same time period, comorbidity of patients starting RTT went up [16]. At the same time, withdrawal of dialysis as reason for death increased from 12.9 to 20.8 % [16], and many patients indicate they regret having started RRT [17].

As reported in many other studies, also in the current cohort, short term survival is abysmal especially in the older age group (>85). Several authors have indicated that not age per se, but rather underlying comorbidity, might be a predictor of poor outcome, and that outcome can be good even in the elderly, provided there are no other comorbidities present [9, 13]. Indeed, comorbidity and functional and cognitive function can vary substantially amongst older patients, rendering age itself less relevant as a discriminating prognostic factor. Also in the risk stratification score of Couchoud et al [15], comorbidity is much more important for prognosis than age, which only contributes an additional 1 or 2 points for those older than 85 or 90 respectively. Nevertheless, in our current validation cohort, it was clear that age and comorbidity were linked, with more than half of the patients older than 85 being in the highest risk stages of the REIN score. However, vice versa, it also indicates that a substantial part of the very old does not have comorbidity, and might thus have a good prognosis on dialysis. Thus, our data confirm that age cannot be the sole criterion to decide to start or withhold dialysis, though older age is often associated with more comorbidities.

The use of a risk stratification score can be very helpful to better illustrate the odds of a good or bad outcome in an objective manner. Results of the score and the associated risk, if presented in a format the patient can understand, can be of real value in guiding a shared decision making by creating realistic expectations about the treatment [18–20]. Our external validation of the REIN risk stratification score corroborates its use for such purposes.

Shared decision making comprises 3 stages: informing the patient, elicit his/her preferences and values, and assist decision making [21]. The REIN score [15] appears to be sufficiently accurate to provide the necessary prognostic information to the patient. Care should be taken however on the way this information is presented to the patient, as most patients and even physicians struggle in interpreting relative risks and statistical data [22, 23]. Easy pictographic presentations as presented in Fig. 2 can be helpful to convey the underlying messages of the data and their implications to the patient. However, patient’s response to and conclusions drawn from the presented data might be different, based on their background beliefs, values and expectations of how their life (or death) should look like. For an 85 year old, even a 50 % probability of mortality at 3 months might still justify start of dialysis when an important event, for example a marriage of a grandchild, is planned in the near future, whereas for another individual, even a 15 % mortality probability at 6 months might not suffice to choose starting or continuing dialysis if the accompanying quality of life is too poor. The elicitation of the patient’s preferences and values is an important part of the shared decision making process, and it is important that the involved professionals avoid mixing up their own beliefs, values and expectations with those of the patient and/or his next of kin. A parallel can be drawn with the area of dialysis withdrawal, where there is evidence that there is substantial peer pressure amongst nephrologists on whether or not they believe that withdrawal of dialysis is ethically defendable [24]. More important, there is a strong correlation between the actual occurrence of dialysis withdrawal in a centre and the beliefs of the treating physician [24]. These observations underscore the need for easy and neutral pictographic presentation of relative risk, minimizing external bias and maximizing patient autonomy.

It can be argued that we, as did the original REIN study, only included patients that actually did start dialysis, and that we therefore do not know the fate of patients that did not start dialysis. Several studies underscore however that in patients with one or more comorbidities, survival is equal in those who did or did not start dialysis. Most patients who opt not to start dialysis most likely have multiple comorbidities, and would thus end up in the higher risk scores. As such, we believe the use of a cohort that actually started dialysis does not jeopardize the conclusion of its validity to identify patients at high risk of poor outcome.

A limitation of our validation might be that the REIN cohort has similar properties in terms of patient characteristics as the NBVN cohort. In addition, REIN and NBVN operate in a comparable healthcare environment in terms of organisation and cultural background. As a consequence, it can still be that the REIN score does not discriminate sufficiently well in other healthcare settings or regions.

The NBVN registry does not capture mobility and need for assistance for transfer, parameters of functional capacity. By consequence, we were not able to capture frailty in our patients. Frailty is a relatively new emerging concept indicating a lack of physiological reserve. It is difficult to define in strict terms, but its presence can easily be assessed from clinical observations. In the REIN risk stratification tool, it is mainly reflected in the “mobility” issue, and it contributes substantially to the overall risk, with 9 points for the maximal score. Different geriatric assessment scores for frailty exist, but most of them are laborious and/or time consuming, and cannot be performed on a regular or repetitive basis. It has been demonstrated before that frailty and classical comorbidity do not always completely overlap [25]. A simple assessment of the REIN score can discriminate those patients who might benefit from a more extensive geriatric assessment by a geriatrician.

It could be argued that the score could be further improved by adding extra elements. However, it has been advocated before that the search for a highly accurate score for use in clinical practice is probably not justified [26]. Indeed, patients rather interpret information provided by the scores in more general qualitative rather than in absolute numerical terms. A more complex score might be cumbersome and time consuming to use in clinical practice. Therefore, absolute accuracy should not be strived for, and ease of use is probably more of importance. One factor that might improve the score is to include mode of vascular access (catheter vs graft vs fistula), as this has been demonstrated to be associated with outcome. However, most likely, there is a high collinearity between the items already included in the score, and the propensity to have a catheter as first access.

## Conclusions

Our paper provides an external validation of a clinically applicable risk stratification tool. Such a tool is crucial to assist evidence based shared decision making on whether to start dialysis or opt for conservative care.

### Ethics approval and consent to participate

This study was approved by the ethical committee of the Ghent University Hospital.

### Consent for publication

As only summary congregated data are presented, consent for publication of patients is not applicable.

### Availability of data and materials

Supplementary data on prognostication at 3, 6 and 12 months are available in “Additional file 1: Table S1”.

A synopsis of the NBVN registry data is available at the NBVN website (www.nbvn.be) under the heading “jaarverslag”.

As far as not already presented in the manuscript or on the website, more detailed data can be obtained after permission to do so is granted by the NBVN registry steering committee. Please send a request to the corresponding author at wim.vanbiesen@ugent.be.

## Additional file

Additional file 1: Table S1. Survival at 3, 6 and 12 months according to aREIN score. Survival (in %) according to aREIN score at 3, 6 and 12 months. (DOCX 10 kb)

## Abbreviations

aREIN: abbreviated REIN score
AUC: area under the curve
BMI: body mass index
CKD: chronic kidney disease
COPD: chronic obstructive pulmonary disease
eGFR: estimated glomerular filtration rate
HD: haemodialysis
MDRD: modification of diet in renal disease
NBVN: Nederlandstalige Vereniging voor Nefrologie
NYHA: New York Heart Association
PCI: Percutaneous Coronary Intervention
PD: Peritoneal Dialysis
REIN: Renal Epidemiology Information Network
RRT: Renal Replacement Therapy
