# Supplementary material for: External Validation of a risk stratification model to assist shared decision making for patients starting renal replacement therapy
Source: BMC Nephrol. 2016 Apr 7;17:41. doi: 10.1186/s12882-016-0253-3 (PMC4823864; doi:10.1186/s12882-016-0253-3)
Supplement: Additional file 1: Table S1. — Survival at 3, 6 and 12 months according to aREIN score. Survival (in %) according to aREIN score at 3, 6 and 12 months. (DOCX 10 kb) [file 12882_2016_253_MOESM1_ESM.docx]

| **aREIN score** | **N** | **3month survival (%** | **6month survival (%)** | **12month survival**  **(%)** |
| --- | --- | --- | --- | --- |
| **0** | **257** | **100** | **98.4** | **96.5** |
| **1** | **343** | **97.4** | **95.9** | **93.9** |
| **2** | **277** | **95.7** | **93.5** | **89.2** |
| **3** | **390** | **96.7** | **93.1** | **88.7** |
| **4** | **256** | **95.3** | **91.4** | **85.2** |
| **5** | **355** | **92.1** | **85.4** | **74.9** |
| **6** | **264** | **90.9** | **82.6** | **72.7** |
| **7** | **182** | **87.4** | **79.1** | **67.0** |
| **8** | **156** | **83.3** | **77.6** | **64.1** |
| **9** | **92** | **76.1** | **65.2** | **52.2** |
| **10** | **72** | **75.0** | **55.6** | **44.4** |
| **11** | **17** | **70.6** | **58.8** | **47.1** |
| **12** | **13** | **69.2** | **38.5** | **23.1** |
| **13** | **4** | **100** | **50** | **25.0** |
